# Inoculations with Arbuscular Mycorrhizal Fungi Increase Vegetable Yields and Decrease Phoxim Concentrations in Carrot and Green Onion and Their Soils

**DOI:** 10.1371/journal.pone.0016949

**Published:** 2011-02-09

**Authors:** Fa Yuan Wang, Rui Jian Tong, Zhao Yong Shi, Xiao Feng Xu, Xin Hua He

**Affiliations:** 1 Agricultural College, Henan University of Science and Technology, Luoyang, China; 2 Department of Life Science, Luoyang Normal University, Luoyang, China; 3 Centre for Ecosystem Management, School of Natural Resources, Edith Cowan University, Joondalup, Austrailia; 4 State Centre of Excellence for Ecohydrology and School of Plant Biology, University of Western Australia, Crawley, Australia; Cinvestav, Mexico

## Abstract

**Background:**

As one of the most widely used organophosphate insecticides in vegetable production, phoxim (C_12_H_15_N_2_O_3_PS) is often found as residues in crops and soils and thus poses a potential threat to public health and environment. Arbuscular mycorrhizal (AM) fungi may make a contribution to the decrease of organophosphate residues in crops and/or the degradation in soils, but such effects remain unknown.

**Methodology/Principal Findings:**

A greenhouse pot experiment studied the influence of AM fungi and phoxim application on the growth of carrot and green onion, and phoxim concentrations in the two vegetables and their soil media. Treatments included three AM fungal inoculations with *Glomus intraradices* BEG 141, *G. mosseae* BEG 167, and a nonmycorrhizal control, and four phoxim application rates (0, 200, 400, 800 mg l^−1^, while 400 mg l^−1^ rate is the recommended dose in the vegetable production system). Carrot and green onion were grown in a greenhouse for 130 d and 150 d. Phoxim solution (100 ml) was poured into each pot around the roots 14d before plant harvest. Results showed that mycorrhizal colonization was higher than 70%, and phoxim application inhibited AM colonization on carrot but not on green onion. Compared with the nonmycorrhizal controls, both shoot and root fresh weights of these two vegetables were significantly increased by AM inoculations irrespective of phoxim application rates. Phoxim concentrations in shoots, roots and soils were increased with the increase of phoxim application rate, but significantly decreased by the AM inoculations. Soil phosphatase activity was enhanced by both AM inocula, but not affected by phoxim application rate. In general, *G. intraradices* BEG 141 had more pronounced effects than *G. mosseae* BEG 167 on the increase of fresh weight production in both carrot and green onion, and the decrease of phoxim concentrations in plants and soils.

**Conclusions/Significance:**

Our results indicate a promising potential of AM fungi for enhancing vegetable production and reducing organophosphorus pesticide residues in plant tissues and their growth media, as well as for the phytoremediation of organophosphorus pesticide-contaminated soils.

## Introduction

Organophosphorus pesticides with higher acute toxicities than the banned organochlorine ones have been major alternatives worldwide for plant pest control in agriculture since 1980’s [1], [2]. At present more than 70% of the total 1.7 million tons pesticides produced in China are organophosphorus ones [1]. The current average pesticide application rate is 15 kg ha^−1^ in China, which is similar to that in Japan (12 kg ha^−1^), but much higher than that in Korea (6.6 kg ha^−1^) and India (0.5 kg ha^−1^) [3]. In general, the chemical residues of organophosphorus pesticides in agricultural products have exceeded the food safety standards in China and hence pose a public health risk to its 1.3 billion consumers [4]–[7]. For example, the maximum residue limits (MRLs) in vegetables is 0.05 µg phoxim (C_12_H_15_N_2_O_3_PS) g^−1^ fresh weight in China. However, the average phoxim residue in vegetable crops has almost doubled that to 0.09 µg g^−1^ fresh weight [8], whilst the acceptable daily intake for the human being is 0–0.001 mg kg^−1^ bodyweight or the LD50 (50% lethal dose) of phoxim for the rat is about 2 g kg^−1^ bodyweight [1]. Furthermore, phoxim is applied to both soil and foliage and also as a seed dressing to control unwanted insects and underground pests in agriculture worldwide including Australia, Egypt, South Africa, Turkey and some European and American countries [9], [10], and up to 10 million kg phoxim per year in China is currently applied [2]. More profoundly, phoxim belongs to plant-metabolized organophosphorus pesticides [11] with an acute toxicity to mammal reproduction [12]–[14], and phoxim and/or its plant metabolites exposure to human beings can cause headache, weakness, giddiness, nausea, vomiting, and stomach pains [10]. If a massive dose has been swallowed, the toxicity can be fatal [15]–[17]. As a consequence of its potential risk to public health through the food chain, studies on reducing the phoxim residues in crop products particularly from high input intensive vegetable systems, as well as in their production environments are urgently needed.

Arbuscular mycorrhizal (AM) fungi are ubiquitous symbiotic associations found in both natural and agricultural ecosystems, including organics-contaminated sites [18]–[20]. AM fungi provide direct links between soil and roots, and consequently may have significant influences on the uptake and translocation of organics by plants, as well as the dissipation and degradation of organics in soils including atrazine [21], [22], PAHs (polycyclic aromatic hydrocarbon) [23]–[26], DDT [27] and weathered *p,p*-DDE in soils [28]. Putative mechanisms involved in interactions between AM fungi and organics include direct effects of enzymes secreted by hyphae, and indirect effects of enhanced root-derived enzymes, microbial activity and modified microbial composition, and unspecific effects of changes in pH, osmotic potential, red-ox potential, partial pressures of O_2_/CO_2_ [22], [26], [27], [29], [30]. Meanwhile, Koide and Kabir [31] found that the extraradical hyphae of *G. intraradices* hydrolysed both 5-bromo-4-chloro-3-indolyl phosphate and phenolphthalein diphosphate. In addition, AM inoculation did increase plant growth whilst phoxim did not decrease the AM colonization of *Cynara cardunculus* [32]. Thus, it is expected that AM fungi make a direct and/or indirect contribution to enhancing the biodegradation of organophosphorus pesticides and consequently decreasing pesticide residues in crops grown in polluted sites. However, to our knowledge, no reports have been studied the relationship between AM fungi and the degradation and residues of organophosphorus pesticides in soils or plants.

We hypothesize that AM inoculation may make a contribution to plant growth and phoxim degradation in a phoxim-contaminated soil, if the applied AM fungi are not sensitive to phoxim. To test our hypothesis with two AM fungal isolates and four phoxim rates, a pot experiment was carried out (1) to assess the effect of AM inoculation on the growth of carrot and green onion grown in soils with different application rates of phoxim; and (2) to identify whether AM inoculation can decrease the phoxim concentrations in the vegetables and their growth media.

## Materials and Methods

### AM inocula and plant seeds

Two AM fungal inocula, *Glomus intraradices* BEG 141 and *G. mosseae* BEG 167, kindly provided by China Agricultural University, were propagated with alfalfa (*Medicago sativa*) in a soil–sand mixture in greenhouse for three successive cycles (four months each). The inocula were air-dried and sieved (2 mm), and each consisted of a mixture of rhizospheric soil containing fine root fragments, hyphae and spores (54 and 68 spores/g air-dried soil for BEG 141 and BEG 167, respectively). The nonmycorrhizal control inoculum was also produced by the same procedure to provide a similar other microbes.

Uniform size seeds of carrot (*Daucus carota* L. cv. Changfeng) and green onion (*Allium fistulosum* L. cv. Fengwang) were surface-sterilized with 10% (v/v) H_2_O_2_ for 10 min, and subsequently washed several times with distilled water.

### Plant growth media

Soils collected from an experimental field (0–15 cm depth) at the Henan University of Science and Technology, were autoclaved at 121°C for 2 h, and air-dried and then thoroughly mixed as the plant growth media. The soil is classified as Aquic Ustochrepts (US soil taxonomy) and soil texture is loamy, with a pH (1∶2.5 soil/water) 7.9, 1.62% organic matter 65.3 mg kg^−1^ alkali-hydrolyzable N, 21.4 mg kg^−1^ Olsen P, and 120.0 mg kg^−1^ 1 M NH_4_OAc extractable K.

### Pesticide, reagents and standards

The phoxim was a commercial 40% phoxim emulsifying concentrate widely used in China. Standard substance of phoxim (0.1 mg ml^−1^) purchased from Beijing Beihua Hengxin Biotechnology Co. Ltd was used for phoxim analysis. Acetonitrile was HPLC-grade and other reagents were analytical grade.

### Experimental procedure

This experiment was a bifactorial design consisting of four phoxim rates (0, 200, 400, 800 mg l^−1^, deionized water diluted from 40% phoxim emulsifying concentrate) and three inoculation treatments (nonmycorrhizal, BEG 141 and BEG 167). The 400 mg l^−1^ phoxim application rate represents the recommended dose that was widely applied in the vegetable production in China. Mycorrhizal inoculation treatments received 20 g mycorrhizal or nonmycorrhizal inocula, which were placed in the middle layer of growth media in pottery pots (1.2 litre volume, 1200 g soil in each pot). As a result, there were 12 treatments with three replicates for each plant species, giving a total of 72 pots in a randomized block arrangement.

Seeds of carrot and green onion were sown separately on July 31, 2008. Two seedlings of carrot and six of green onion were left after emergence and grown in a non-environment controlled greenhouse on the campus of the Henan University of Science and Technology. Plants were irrigated with tap water to maintain about 70% of filed water holding capacity and no fertilizers were supplied in the whole experiment.

Phoxim is usually applied at any growth period of a vegetable in Chinese vegetable production system, but a high chemical residue is observed if applied at the harvest period. Thus, 100 ml phoxim solution was slowly poured into the growth soil of each pot around the roots of the carrot and green onion 14 d before harvest. Pots were left without watering for 2 d prior to the phoxim application. For the nonphoxim treatment, 100 ml deionized water was added. The phoxim applied area was covered with a 0.5 cm depth of soil.

Carrot and green onion were respectively harvested after 130 d and 150 d of growth. Shoots and roots were separated, rinsed with distilled water, wiped with tissue paper, and weighed immediately. A portion of fresh fibrous roots of carrot and green onion roots were used to assess mycorrhizal colonization, and the remaining roots were stored at −20°C for phoxim analysis. Soils from each pot were thoroughly mixed after root harvest and 100 g fresh soils were stored at −20°C for phoxim analysis. The remaining soils were air-dried for phosphatase activity or oven-dried for water content (%) analysis.

### Plant and soil analysis

Determination of root mycorrhizal colonization was followed by the acid fuchsin staining-grid intersect method [33]. Soil phosphatase activity was determined by measuring the *p*-nitrophenol (PNP) released by phosphatase when soils were incubated with buffered (pH 7.0) sodium *p*-nitrophenyl phosphate solution and toluene at 37°C for 24 h [34].

Analysis of phoxim concentrations in shoots, roots and soils was followed by the Ministry of Agriculture of China [35] and the national standard of China (GB/T 14552-2003) [36], using an Agilent 1100 HPLC system (Agilent, USA) equipped with an auto-injector and a 280 nm DAD detector. An Agilent ZORBAX SB-C18 column (150 mm×2.1 mm i.d., 5 µm, Agilent, Santa Clara, CA, USA) was used for separations. The mobile phase was a mixture of acetonitrile–water (65∶35, V/V), and the flow rate was 0.45 ml min^−1^. The column temperature was set at 30°C, and the injection volume was 4 µl. The recoveries were between 95.0% and 100.4%.

### Statistical analysis

Data (means ± SE, *n* = 3) were subjected to a two-way ANOVA using the SPSS 13.0 software package (SPSS Inc., Chicago, USA). Significances between the means under AM inoculation, phoxim application rate, or interactions of AM inoculation and phoxim application were compared by the Duncan’s multiple range test at *p*<0.05, 0.01 or 0.001.

## Results

### Mycorrhizal colonization

**Figure 1:**
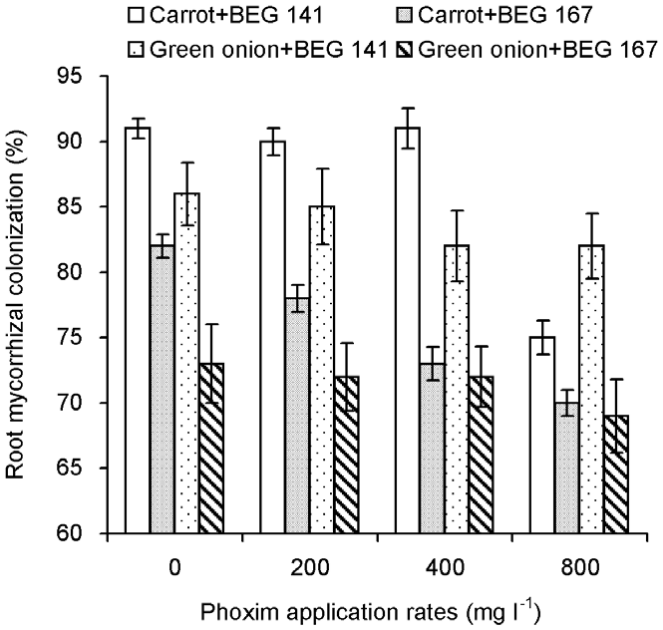
Root mycorrhizal colonization (%) (means ± SE, *n* = 3) of carrot and green onion. BEG 141 and BEG 167: *G. intraradices* BEG 141 and *G. mosseae* BEG 167, respectively.

Plants in the nonmycorrhizal controls were not colonized. Percentage of root mycorrhizal colonization of both carrot and green onion was higher than 70%, but lower when inoculated with BEG 167 than with BEG BEG 141 (Figure 1). Root mycorrhizal colonization showed a decreasing trend with the increase of phoxim application rate, and significantly decreased in carrot, but not in green onion, when 800 phoxim mg l^−1^ was applied. Two-way ANOVA results show that AM inoculation, phoxim application, and the interactions between AM inoculation and phoxim application all had significant effects on mycorrhizal colonization for carrot. In contrast, only AM inoculation had significant effect on mycorrhizal colonization for green onion (Table 1).

**Table 1:** Significance levels (*F*-values) of treatments and treatment interactions on measured variables on a two-way ANOVA analysis.

Variables	AM inoculation		Phoxim application		AM × Phoxim	
	Carrot	Green onion	Carrot	Green onion	Carrot	Green onion
Mycorrhizal colonization	248.9***	212.6***	64.3***	ns	13.3***	ns
Shoot fresh weight	253.5***	138.6***	5.7*	ns	6.9*	ns
Root fresh weight	288.6***	245.2***	ns	ns	4.0*	ns
Shoot phoxim concentration	284.7***	154.3***	412.0***	403.5***	60.2***	35.1***
Root phoxim concentration	195.8***	165.3***	257.9***	189.6***	52.1***	22.3***
Soil phoxim concentration	156.3***	102.5***	322.8***	308.7***	44.4***	35.6***
Soil phosphatase activity	197.4***	202.1***	ns	ns	ns	ns

Significant levels:

*p<0.05,

**p<0.01,

***p<0.001; ns, non-significant effect.

### Plant fresh weights

**Figure 2:**
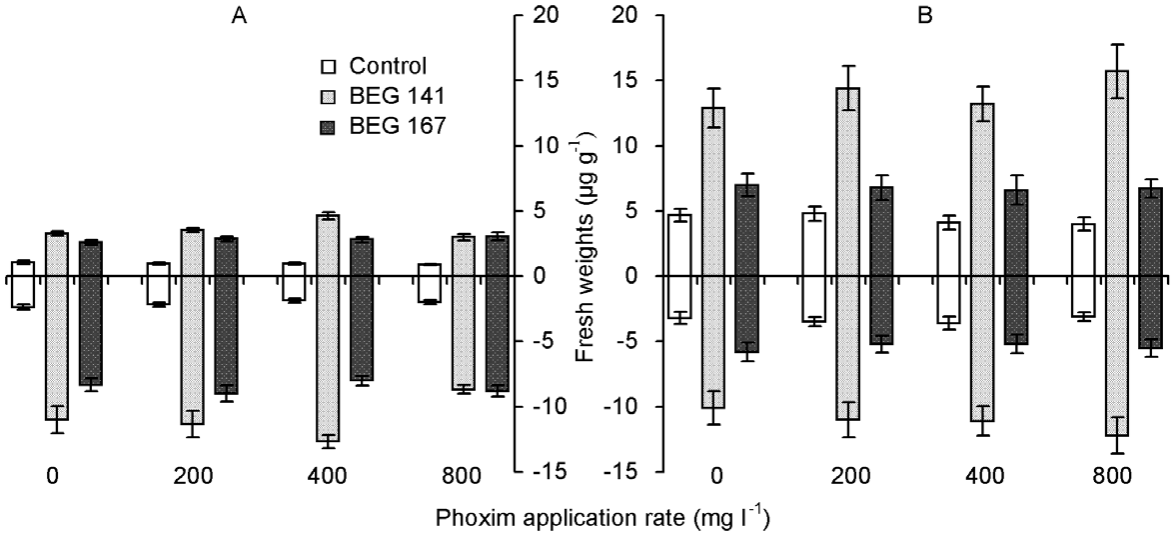
Shoot (above X-axis) and root (below X-axis) fresh weights (means ± SE, *n* = 3) of carrot (A) and green onion (B). Control, BEG 141 and BEG 167: non-AM-inoculation control, *G. intraradices* BEG 141 and *G. mosseae* BEG 167, respectively.

Compared to the non-AM-inoculation control, both BEG 141 and BEG 167 significantly increased carrot fresh weights at all phoxim application rates, and the average yields of carrot roots (edible part) in the BEG 141 and BEG 167 treatment were 5.2 and 4.1 times, respectively (Figure 2a, Table 1). Phoxim application rates did not affect shoot or root fresh weights of carrot in the nonmycorrhizal control and BEG 167 treatments. However, a significantly higher shoot fresh weight in the BEG 141 treatment was observed under the 400 mg l^−1^ phoxim application rate.

Phoxim application did not affect shoot or root fresh weights of green onion (Figure 2b, Table 1). Both AM inocula significantly enhanced the shoot and root fresh weights of green onion, and BEG 141 was more effective than BEG 167 (Figure 2b, Table 1). The average shoot (edible part) yields of green onion in the BEG 141 and BEG 167 treatment were 3.3 and 1.5 times of those of the nonmycorrhizal controls, respectively.

Two-way ANOVA results show that the interactions between AM inoculation and phoxim application had significant effects on both shoot and root biomass production of carrot, but not on shoot or root biomass production of green onion (Table 1).

### Phoxim concentrations in plants

**Figure 3:**
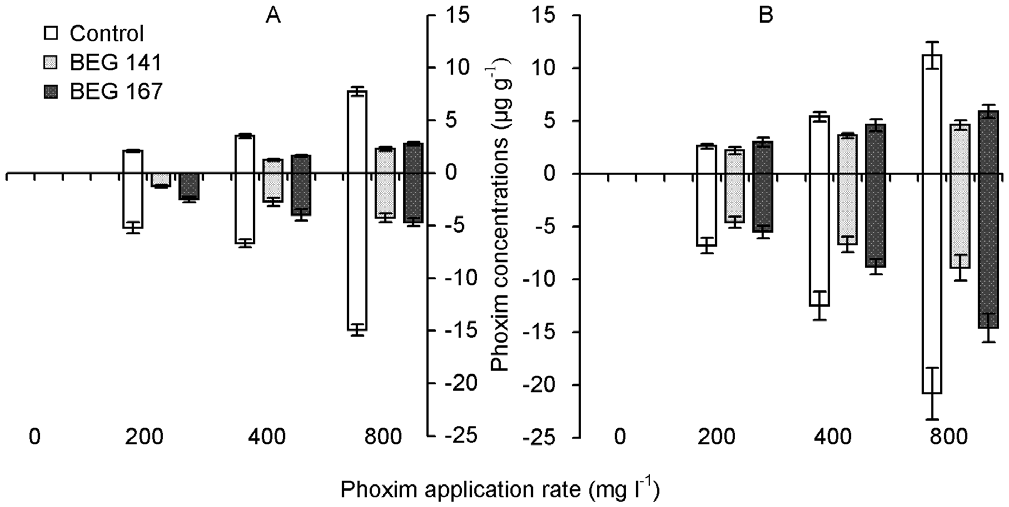
Shoot (above X-axis) and root (below X-axis) phoxim concentrations (means ± SE, *n* = 3) of carrot (A) and green onion (B). Control, BEG 141 and BEG 167: non-AM-inoculation control, *G. intraradices* BEG 141 and *G. mosseae* BEG 167, respectively.

Phoxim was not detected in shoots and roots of both carrot and green onion under the zero phoxim application, whilst increased with the increase of phoxim application rate (Figure 3). For both carrot and green onion, phoxim concentrations were higher in the roots than in the shoots, and both AM inocula significantly decreased phoxim concentrations in either shoots or roots. In addition, phoxim concentrations in both shoots and roots were lower under the BEG 141 than under the BEG 167 inoculation (except for in carrot root at 800 mg l^−1^ phoxim application rate).

All treatments with AM inoculation or phoxim application rate, and the interactions between AM inoculation and phoxim application all had significant effects on phoxim concentrations in shoots and roots for both carrot and green onion (Table 1). In addition, the effects of AM inoculation were more pronounced on phoxim concentrations in carrot than in green onion.

### Phoxim concentrations in soil

**Figure 4:**
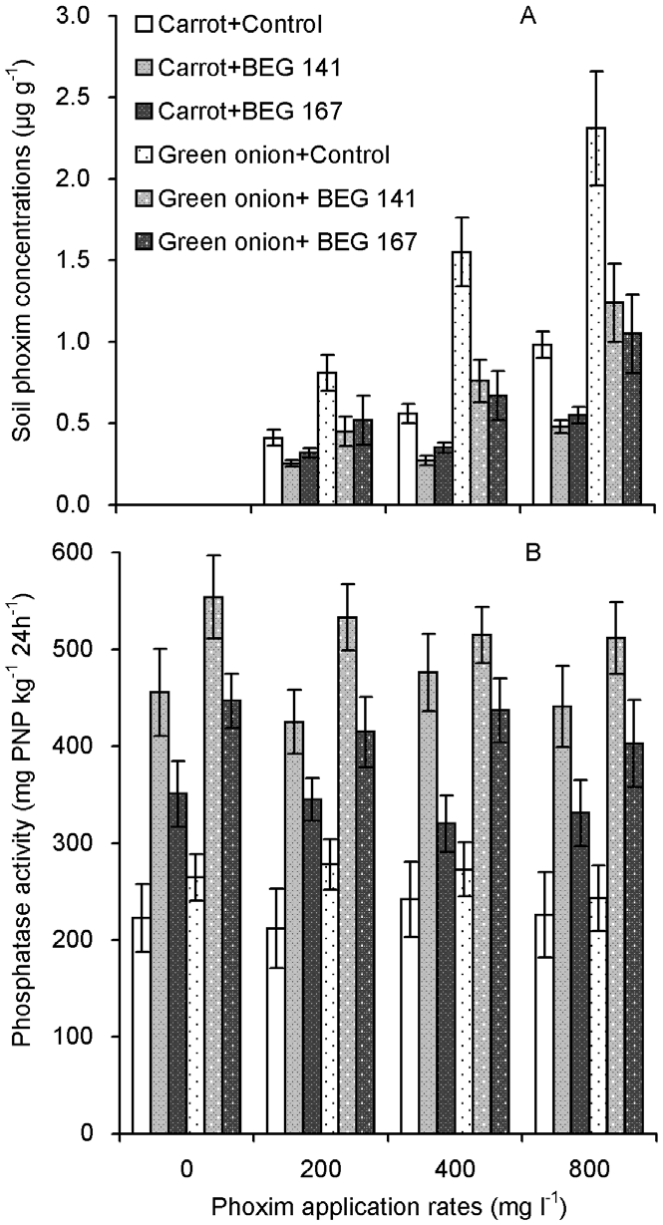
Soil phoxim concentrations (A) and phosphatase activity (B) (means ± SE, *n* = 3) after carrot and green onion harvest. Control, BEG 141 and BEG 167: non-AM-inoculation, *G. intraradices* BEG 141 and *G. mosseae* BEG 167, respectively.

Phoxim was not detected in the soil growth media under the zero phoxim application, whilst increased as the phoxim application rate increased (Figure 4a). Both AM inocula significantly decreased phoxim concentrations in the soil collected from both the carrot and green onion pots, and BEG 141 showed a stronger effect on the decrease of phoxim concentration than BEG 167 for the carrot, but not for the green onion.

Two-way ANOVA results show AM inoculation, phoxim application, and the interactions between them all had significant effects on phoxim concentrations in soils from both the carrot and green onion pots (Table 1).

### Soil phosphatase activity

Soil phosphatase activity did not vary with the increase of phoxim application rate (Figure 4b). Compared with the nonmycorrhizal controls, soil phosphatase activity in the carrot and green onion pots was significantly increased by these two AM inocula, and the BEG 141 had a stronger positive effect than the BEG 167. In addition, soil phosphatase activity was generally lower in the carrot pots than in the green onion pots.

Soil phosphatase activity was significant affected by the AM inoculation, but not by the phoxim application. In addition, no significant interactions were observed between the AM inoculation and phoxim application rate on the soil phosphatase activity (Table 1).

## Discussion

Numerous studies have reported that organophosphorus pesticides at recommended dosages have little or no adverse effects on AM colonization and host plant growth [11], [37]–[41], but at higher dosages generally show deleterious effects [38], [40]. For example, phoxim applied at the recommended dose had no significantly adverse effects on AM colonization and the growth of *Cynara cardunculus* [11]. Our results show that phoxim had no adverse effects on the growth of these two vegetables or soil phosphatase activity (Fig. 4), and only 800 mg l^−1^ phoxim (2 times of the recommended dosage) inhibited mycorrhizal colonization of carrot. These results indicate that phoxim is generally of low toxicity to AM fungi and the formation of AM symbiosis, and the recommended dosage of phoxim (400 mg l^−1^) is safe to AM fungi and plant growth.

Obviously, the effects of pesticides on AM fungi vary with their toxicity and application rates, the tolerance of AM fungi, host plants, and environmental factors. AM fungi react differently to pesticides [11], [41], [42]. In the present study, *G. intraradices* BEG 141 showed more pronounced effects than *G. mosseae* BEG 167 on both the growth of vegetables and phoxim residues in plants and soils, probably because of a high colonization potential of *G. intraradices* BEG 141 and a high compatibility between this isolate and the two vegetables tested. Also, the two AM inocula were more effective on carrot than on green onion. This may be due to different mycorrhizal dependency of plants and soil P nutrition needed by plants. Mycorrhizal development is generally reduced under high soil P, and in general the mycorrhizal effects on plant growth occur significantly in low P soils. Although green onion was also easily colonized, the available soil P (21.4 mg kg^−1^) in the present study might be high for this plant and the mycorrhizal effects were thus decreased. In contrast, carrot is one of the plants with high mycorrhizal dependency, and high soil P (100 mg kg^−1^) is considered as low for this crop [43], and the low available P in this present experiment would thus enhance mycorrhizal effects on carrot.

The mechanisms behind the decreased phoxim concentrations by AM inoculation may be diverse. One possible reason is the dilution effect caused by an enhanced plant growth due to an improved mineral nutrition especially P uptake, which may partly explain the lower phoxim concentrations in mycorrhizal plants. However, the dilution effect was unlikely to be the only explanation, because mycorrhizal roots had higher biomass and larger surface area, and if they have the same or higher capacity for phoxim uptake, the phoxim concentrations in their tissues should not be lower than in the nonmycorrhizal controls. In our results, phoxim concentrations in roots and shoots were all much lower in the mycorrhizal than in the nonmycorrhizal treatments (Figure 3). Additionally, phoxim concentrations in the soil were also decreased by the AM inoculation (Figure 4a). Therefore, there must be other mechanistic explanations. Joner and Leyval [30] summarized two main indirect effects involved in the mycorrhizal effects on PAH degradation including enhanced microbial degradation activity and enhanced enzyme activity in roots and rhizosphere soils, which have been proved in recent studies [22], [26], [27]. Our present results also showed the positive mycorrhizal effects on soil phosphatase activity. Hence, AM fungi may help to degrade phoxim via similar mechanisms.

AM fungi are believed to have no direct catabolism or cooxidation on pollutants such as PAH, because they have very limited saprophytic capacities [30]. However, some AM fungi can both enhance the decomposition of complex organic material (grass leaves) in soils and thus increase N capture, indicating that AM symbiosis can have saprotrophic capability [44]. Numerous studies have found that mycorrhizal hyphae can produce phosphatase to hydrolyse organic P compounds and hence contribute to the utilization of P by plants [45]–[48]. Using a split-dish *in vitro* carrot mycorrhiza system free from contaminating microorganisms, Koide and Kabir [31] found that extraradical hyphae of *G*. *intraradices* can hydrolyse organic P, and that the resultant inorganic P can be taken up and transported to host roots. On the other hand, phoxim can be degraded and utilized by bacteria [49]–[51] and fungi such as *Paecilomyces lilaciuns* [52] as an organic source of P and N. Thus, it might be inferred that AM fungi play a direct role in the catabolic process of phoxim in soil.

Phoxim is one of plant-metabolized organophosphorus insecticides [11]. AM fungi may indirectly influence its metabolic process in plants through mycorrhizal effects on plant metabolic activities. For example, AM fungi can colonize root cells to form arbuscules and vesicles, and thereby may directly participate in phoxim metabolism and/or cooperate with plants. Moreover, only the molecular form of phoxim was determined in the present experiment, and the metabolic intermediates were not included but may be present in the soil, plants and AM fungi. Future studies using ^14^C, ^15^N or ^32^P-labelled phoxim should be carried out to study the metabolic pathway and the fate of this pesticide in AM fungal structures including mycelia and spores. Analyses of AM fungal gene sequences coding for pesticide-degrading enzymes may provide direct evidences on whether AM fungi can degrade this pesticide. In addition, it would be interesting to investigate if sulfate (S) could also be released from the phoxim since S plays a role in the pungency of green onion.

Our results have provided the evidence that AM fungi simultaneously increase vegetable yields and decrease the concentrations of organophosphorus pesticides in both plants and soils, but the effects vary with AM fungal species, host plants and pesticide application rate. Our results indicate a promising potential of AM fungi for reducing the accumulation of pesticide in vegetable crops and for the phytoremediation of pesticide-contaminated soils, although such effects are needed to be further studied. It is interesting to note that AM fungi can also enhance the tolerance of host plants to pathogens [53], and thus decrease the amount and frequency of pesticides application, and improve crop quality. In this context, it would be more valuable to study the relationships between more pesticides and further combinations of AM fungi and host plants.
